# Correspondence on Orthodontia

**Published:** 1905-07

**Authors:** E. A. Bogue

**Affiliations:** 63 West Forty-Eighth Street, New York City


### Domestic Correspondence.

### CORRESPONDENCE ON ORTHODONTIA.

June 6, 1905.

To the Editor:

Sir,—I send you a little bit of a correspondence that has recently passed between one of my most respected fellow-practitioners and myself.

The end is not yet, but it occurs to me that a good many of your readers may be interested in the question raised and in the answer that may perhaps be elicited from any one who knows how to surmount the difficulty mentioned.

I should esteem it a great favor to receive an adequate reply to my question.

Truly yours,

E. A. Bogue.

63 West Forty-eighth Street, New York City.

Dear Doctor,—What do you do in cases of unilateral dislocation of the principal molars?

How do you correct the misplaced teeth without interfering with the others?

*Answer:* "It is difficult to answer the question regarding unilateral dislocation. I probably do as you do—the best I can—under the varying circumstances.

"Sometimes I have helped matters by using an intermaxillary elastic on one side only; sometimes, perhaps, an extraction might be resorted to."

I wanted so much to talk over with you the actual fundamental principles that underlie orthodontia.

For twenty-three years I have eschewed the extraction of teeth for that purpose.

There has been in that length of time but one exception to this rule in my own practice, and that was a case where a number of teeth had already been lost, and the patient, a young lady, was about thirty years of age.

I have seen one other case (Baker's patient), in which I consented, *at that time*, that extraction was best; but I must be permitted, in the interests of consistency, to claim the right to change my mind when I learn that I have been wrong; and I think I have learned a little every year.

I have lately spent a week in Washington, studying every single one of the children's skulls in the National Museum, and a good many of the adult ones.

I find that when Bonwill's old method of laying out the teeth is followed, there are just enough to go round, no more, no less. That when we get hold of a young patient, especially during the second period of growth (between seven and fourteen), we have occasion, in pretty much all the cases of misplacement of the teeth, to enlarge one or both of the arches.

I did not think this was so universally true until you yourself brought it to my attention.

I find that when we enlarge these arches, especially the upper, we necessarily remove an obstruction that has prevented their proper development. That when this obstruction is removed, nature resumes her work and growth or development begins again; and if permitted or aided, by keeping obstructions out of the way as fast as Nature grows up to them, the whole series of bones lying above and adjacent to the maxillary bone grow up to their normal size.

This enlarges the whole facial region; it enlarges the nasal meatus, the antrum of Highmore, all the sinuses of the ethmoid bone, and straightens the nasal septum, develops the vomer, and provides a normal palatine vault in which the functions of the tongue are freely and easily performed in the enclosure formed by the normal arrangement of all the teeth properly articulating each with the others, according to the arrangement of Nature, which has provided teeth for each individual case exactly adapted to the size of that individual.

The absence, therefore, from whatever cause,—non-eruption, extraction, or accident,—of one single member from the arch completely disarranges, though with more or less grave results, the entire adaptation of the arches to each other. (See page 13 of my paper on Extraction.)

I have laid emphasis upon beginning the work of regulation early, because by that means we can push the completed crowns (by gentle pressure) into their proper places, that the formation of the roots may be completed after they are in place and that the alveolus may form around those roots while these latter are in process of formation.

This, you see, permits all of the teeth to interdigitate or cusp properly; which condition holds them in position, with the assistance of the tongue on one side and the lips and cheeks on the other, through life.

But without this cusping there can be no surety of teeth, moved from one position to another, retaining their new positions.

Yours truly,

E. A. Bogue.

